# Association between female hormone intake and circadian syndrome: A cross-sectional study based on NHANES 2005–2018

**DOI:** 10.1097/MD.0000000000050708

**Published:** 2026-09-11

**Authors:** Zhiqin Ding, Hongqin Ding, Hao Wu, Dan Zheng, Yucheng Liu, Wei Wang, Weifu Xiong

**Affiliations:** a The Third Affiliated Hospital of Nanchang University (The First Hospital of Nanchang), Nanchang, Jiangxi, China; b Hospital of Jiangxi Provincial Corps, Chinese People’s Armed Police Force, Nanchang, Jiangxi, China; c School of Public Health, Nanchang University, Nanchang, Jiangxi, China; d Nanchang University, Nanchang, Jiangxi, China.

**Keywords:** circadian syndrome, cross-sectional study, female hormone, NHANES, restricted cubic spline

## Abstract

Circadian syndrome (CircS) is a newly identified condition that extends the concept of metabolic syndrome (MetS) by incorporating short sleep duration and depression, comprising 7 components. Although the interactions between female hormones and the circadian system have been documented, the exact relationship between hormonal factors and CircS requires further investigation. Data from the National Health and Nutrition Examination Survey spanning 2005 to 2018 were analyzed. A total of 7279 nonpregnant women aged ≥ 20 years were included. Weighted logistic regression was employed to assess the association between female hormone intake and CircS. Additionally, restricted cubic spline regression was utilized to investigate the nonlinear relationships of age and body mass index (BMI) with CircS. Subgroup analyses were conducted to evaluate the consistency of these associations. The weighted prevalence of CircS was 54.4%. In the fully adjusted model, female hormone intake was significantly associated with increased odds of CircS, with an odds ratio (OR) of 1.47 and a 95% confidence interval (CI) ranging from 1.20 to 1.80 (*P* < .001). Restricted cubic spline analysis identified significant nonlinear associations between age and BMI with CircS (*P* < .001), with inflection points at 61.0 years and 29.9 kg/m^2^, respectively. For individuals aged < 61.0 years, each additional year was associated with an 8% increase in the risk of CircS (OR = 1.08, *P* < .001); beyond this age, the risk continued to rise, albeit more slowly (OR = 1.02, *P* = .005). Regarding BMI, for values ≤ 29.9 kg/m^2^, each additional unit was associated with a 33% increase in risk (OR = 1.33, *P* < .001), whereas an 8% increase in risk was observed beyond this threshold (OR = 1.08, *P* < .001). Subgroup analyses showed consistent associations across all strata (all *P* for interaction > .05). Female hormone intake is independently associated with an increased risk of CircS. Age and BMI exhibited nonlinear associations with CircS, with inflection points at 61.0 years and 29.9 kg/m^2^, respectively.

## 1. Introduction

Circadian rhythms are fundamental regulatory mechanisms that maintain physiological homeostasis in mammals. These rhythms are coordinated by the central clock situated in the suprachiasmatic nucleus of the hypothalamus and by peripheral clocks distributed throughout various organs.^[1]^ They play crucial roles in the regulation of sleep–wake cycles, metabolism, hormone secretion, and immune function. Circadian rhythm disruption is strongly associated with several chronic diseases, including type 2 diabetes mellitus, cardiovascular disease (CVD), and metabolic syndrome (MetS).^[2,3]^

MetS is widely used in clinical practice;^[4]^ however, its efficacy in predicting CVD remains unclear. In 2005, the American Diabetes Association and the European Association for the Study of Diabetes issued a joint statement indicating that MetS has been defined imprecisely, casting doubt on its utility as a CVD risk marker.^[5]^ In response, Zimmet proposed the concept of circadian syndrome (CircS), which incorporates short sleep duration and depression into the 5 existing MetS components, resulting in a total of 7 components.^[6]^ This framework underscores the significance of circadian disruption in the onset and progression of metabolic diseases. Numerous studies have shown that CircS is a superior predictor of CVD compared to MetS and is also linked to cognitive impairment and psoriasis.^[7–9]^

Estrogen, beyond its role in regulating female reproductive functions, significantly impacts metabolism, inflammation, and the circadian system.^[10]^ It modulates the expression of CLOCK genes through estrogen receptors, thereby influencing circadian oscillations.^[11]^ Exogenous estrogens, including oral contraceptives and hormone replacement therapy, are extensively utilized worldwide.^[12,13]^ Prior research has demonstrated a close relationship between estrogens and insulin resistance, fat distribution, and inflammatory levels,^[14,15]^ which are fundamental pathophysiological components of CircS. However, no study has directly evaluated the relationship between female hormone intake and CircS.

This study utilized the National Health and Nutrition Examination Survey (NHANES) data from 2005 to 2018 to achieve 3 objectives: to examine the independent association between female hormone intake and CircS; to analyze the nonlinear relationships of age and body mass index (BMI) with CircS; and to assess the consistency of these associations across various subgroups.

## 2. Methods

### 2.1. Study participants

**Figure 1.**
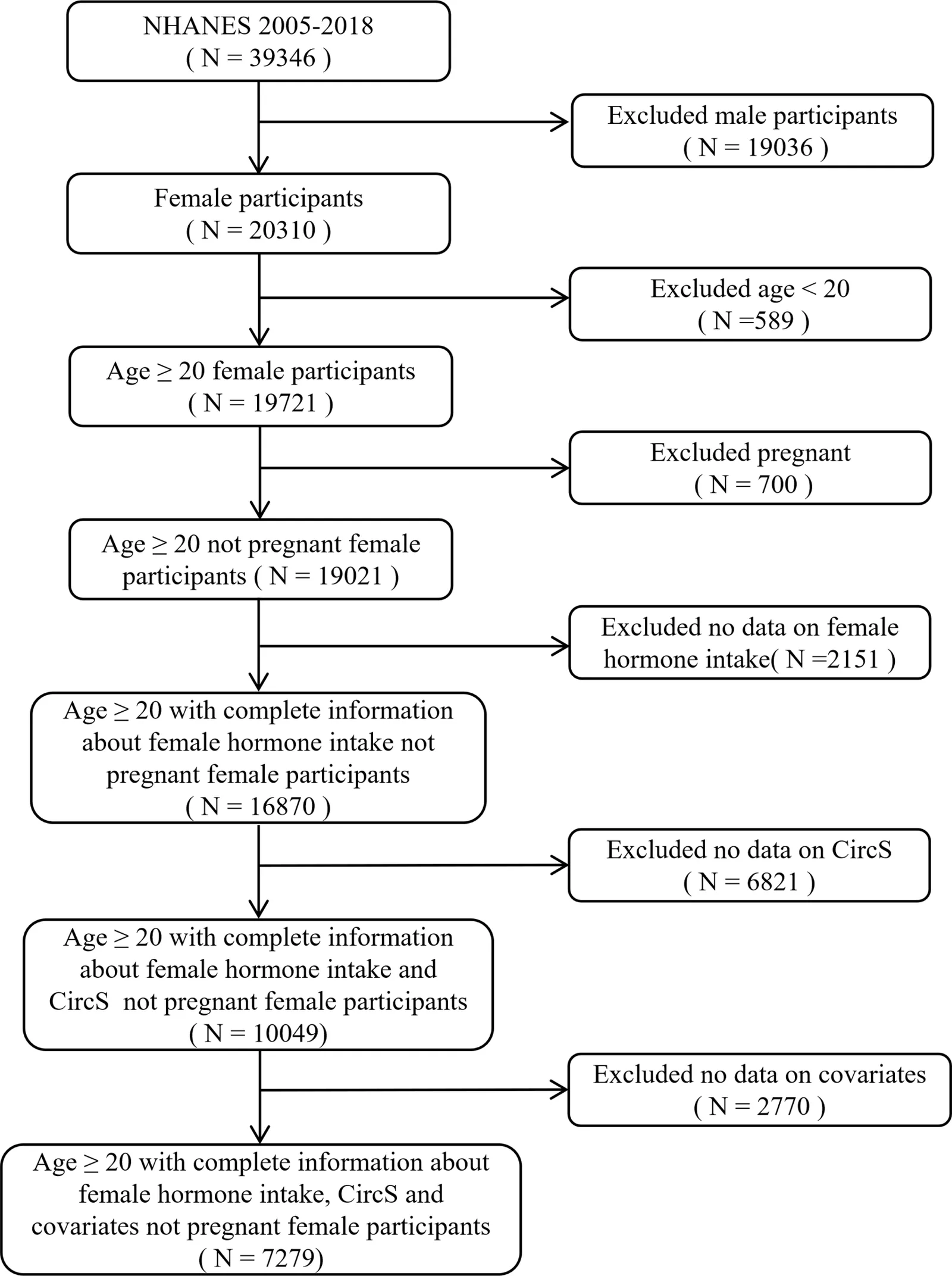
Flowchart of the screening process for participants in NHANES 2005–2018. CircS = circadian syndrome, NHANES = The National Health and Nutrition Examination Survey.

The data utilized in this study were obtained from the NHANES, encompassing 7 cycles: 2005–2006, 2007–2008, 2009–2010, 2011–2012, 2013–2014, 2015–2016, and 2017–2018. Written informed consent was obtained from all participants, and the NHANES protocol was approved by the National Center for Health Statistics Research Ethics Review Board. Initially, the study involved 39,346 individuals. Following the exclusion of males (n = 19,036), individuals under 20 years of age (n = 589), pregnant women (n = 700), participants with missing data on female hormone intake (n = 2151), those lacking CircS data (n = 6821), and those with incomplete covariate information (n = 2770), the final analysis included 7279 participants (Fig. 1).

### 2.2. Exposure and outcome

The exposure variable, defined as the intake of female hormones, was characterized by a positive response to the question: “Have you ever used female hormones such as estrogen and progesterone (include any form of female hormones, such as pills, cream, patch, and injectables, but do not include birth control methods or use for infertility)?” Or the answer is “yes” to the question “Have you ever taken birth control pills for any reason?” According to the existing literature, the outcome variable CircS is characterized by the presence of at least 4 of the 7 specified components. Table 1 provides definitions for each component of MetS and CircS.

**Table 1:** Indicators for metabolic syndrome and circadian syndrome in women.

Indicators	MetS	CircS
Elevated waist circumference was defined as waist circumference ≥ 88 cm	√	√
Elevated fasting glucose was defined as fasting glucose ≥ 100 mg/dL, or drug treatment for elevated glucose	√	√
Elevated blood pressure was defined as SBP ≥ 130 mm Hg or a DBP ≥ 85 mm Hg, or drug treatment for hypertension	√	√
Reduced HDL-C was defined as serum HDL-C < 50 mg/dL in women, or drug treatment for dyslipidemia	√	√
Elevated triglyceride was defined as serum triglycerides ≥ 150 mg/dL, or drug treatment for dyslipidemia	√	√
Short sleep was defined as a sleep duration of < 6 h/d		√
Depression symptoms were measured using PHQ-9. This 9-item instrument is designed to screen for the presence of depression symptoms experienced within the past 2 wks. Participants with a PHQ-9 score *≥ *5 were defined as having depression symptoms		√

CircS = circadian syndrome, DBP = diastolic blood pressure, HDL-C = high-density lipoprotein cholesterol, MetS = metabolic syndrome, PHQ-9 = Patient Health Questionnaire, SBP = systolic blood pressure.

### 2.3. Covariates

Covariates included age, race (Mexican American, Other Hispanic, Non-Hispanic White, Non-Hispanic Black, and Other Race), education level (high school or below, some college, and college graduate or above), marital status (married/living with partner and not married), ratio of family income to poverty (PIR: ≤1.3, 1.3–3.5, and ≥3.5), BMI (≤25, 25–30, and ≥30 kg/m^2^), alcohol use (yes/no, defined as having consumed alcohol in the past 12 months), smoking status (yes/no, defined as having smoked at least 100 cigarettes in lifetime), heart failure (yes/no), coronary heart disease (yes/no), and stroke (yes/no).

### 2.4. Statistical analysis

Continuous variables are expressed as weighted means ± standard errors, while categorical variables are represented as weighted percentages (%). Comparisons between groups were conducted using weighted *t*-tests for continuous variables and weighted chi-square tests for categorical variables.

To evaluate the relationship between female hormone intake and CircS, weighted logistic regression was employed across 3 models. Model 1 remained unadjusted, while Model 2 incorporated adjustments for age, race, education level, marital status, and PIR. Model 3 further adjusted for BMI, alcohol use, smoking, heart failure, coronary heart disease, and stroke. The nonlinear associations of age and BMI with CircS were characterized using restricted cubic spline (RCS) regression with 4 knots (at the 5th, 35th, 65th, and 95th percentiles). Inflection points were identified through piecewise regression. A sensitivity analysis was performed to evaluate the reliability of the results by refitting the fully adjusted model (Model 3) after excluding participants who reported heart failure, coronary heart disease, or stroke at baseline. Subgroup analyses were conducted by stratifying the fully adjusted model according to age, race, education level, marital status, PIR, BMI, alcohol use, and smoking.

All analyses accounted for the complex survey design of NHANES. Statistical analyses were performed using R (http://www.R-project.org, version 4.6.0). A two-sided *P* < .05 was considered statistically significant.

## 3. Results

### 3.1. Characteristics of participants

Table 2 presents the survey-weighted characteristics of the study population. The weighted mean age was 50.47 ± 0.31 years. Among the participants, 4283 individuals had CircS, resulting in a weighted prevalence of 54.4%. The mean age within the CircS group was 56.69 ± 0.34 years, compared to 43.05 ± 0.39 years in the non-CircS group. Within the CircS group, 44.4% of participants possessed a high school education or below, in contrast to 27.5% in the non-CircS group. Individuals who were married or cohabiting constituted 55.9% of the CircS group, compared to 63.1% in the non-CircS group. The average PIR was 2.73 in the CircS group and 3.24 in the non-CircS group. The mean BMI in the CircS group was 33.27 kg/m^2^, with a greater proportion of participants having a BMI of ≥ 30 kg/m^2^ (61.8%) compared to those with a BMI of 25–30 kg/m^2^ (27.1%). The CircS group comprised 51.4% smokers and 73.2% alcohol users, with prevalences of 4.4%, 5.6%, and 6.1% for heart failure, coronary heart disease, and stroke, respectively. All differences were statistically significant (all *P* < .05). However, there was no significant difference in female hormone intake between the groups (*P* = .29).

**Table 2:** Characteristics of participants by categories of circadian syndrome: NHANES 2005–2018.

Variables	Total	Non-CircS	CircS	*P*
N	7279	2996	4283	
Age, Mean (SE)	50.47 (0.31)	43.05 (0.39)	56.69 (0.34)	<.001
Age, % (SE)				<.001
20–34	21.2 (0.7)	35.7 (1.2)	9.1 (0.6)	
35–49	25.3 (0.7)	30.9 (1.2)	20.7 (0.8)	
50–64	30.8 (0.8)	22.8 (1.1)	37.4 (1)	
≥65	22.7 (0.7)	10.6 (0.7)	32.8 (1)	
Race, % (SE)				.005
Mexican American	6.4 (0.5)	6.5 (0.6)	6.2 (0.6)	
Other Hispanic	4.6 (0.4)	4.9 (0.5)	4.3 (0.4)	
Non-Hispanic White	72.2 (1.2)	73.1 (1.3)	71.4 (1.4)	
Non-Hispanic Black	11 (0.8)	9.5 (0.7)	12.3 (0.9)	
Other Race	5.9 (0.4)	6 (0.5)	5.8 (0.5)	
Education, % (SE)				<.001
High school or below	36.7 (1)	27.5 (1.3)	44.4 (1.3)	
Some college	34.9 (0.8)	34.2 (1.1)	35.4 (1.1)	
College grad or above	28.4 (1.1)	38.3 (1.4)	20.2 (1.1)	
Marital status, % (SE)				<.001
Married/Living with partner	59.2 (0.9)	63.1 (1.2)	55.9 (1.1)	
Not married	40.8 (0.9)	36.9 (1.2)	44.1 (1.1)	
PIR, Mean (SE)	2.97 (0.04)	3.24 (0.04)	2.73 (0.05)	<.001
PIR, % (SE)				<.001
≤1.3	21.7 (0.8)	17.6 (0.9)	25.1 (1.1)	
1.3–3.5	36.8 (0.9)	33 (1.2)	39.9 (1.1)	
≥3.5	41.6 (1.1)	49.4 (1.4)	35 (1.4)	
BMI, Mean (SE)	30.29 (0.15)	26.74 (0.16)	33.27 (0.19)	<.001
BMI, % (SE)				<.001
≤25	27.9 (0.8)	48 (1.1)	11.1 (0.6)	
25–30	27.7 (0.7)	28.3 (1)	27.1 (1.1)	
≥30	44.4 (0.9)	23.6 (1.1)	61.8 (1.1)	
Smoking				<.001
No	54 (0.9)	60.3 (1.3)	48.6 (1.1)	
Yes	46 (0.9)	39.7 (1.3)	51.4 (1.1)	
Alcohol use				<.001
No	20.5 (0.7)	13 (0.7)	26.8 (0.9)	
Yes	79.5 (0.7)	87 (0.7)	73.2 (0.9)	
Female hormone intake				.290
No	17.1 (0.7)	17.7 (0.8)	16.6 (0.9)	
Yes	82.9 (0.7)	82.3 (0.8)	83.4 (0.9)	
Heart failure				<.001
No	97.3 (0.2)	99.4 (0.2)	95.6 (0.3)	
Yes	2.7 (0.2)	0.6 (0.2)	4.4 (0.3)	
Coronary heart disease				<.001
No	96.7 (0.3)	99.4 (0.2)	94.4 (0.5)	
Yes	3.3 (0.3)	0.6 (0.2)	5.6 (0.5)	
Stroke				<.001
No	96.1 (0.2)	98.8 (0.2)	93.9 (0.4)	
Yes	3.9 (0.2)	1.2 (0.2)	6.1 (0.4)	

% (SE) = weighted percentage (standard error), BMI = body mass index, CircS = circadian syndrome, Mean (SE) = mean (standard error), NHANES = The National Health and Nutrition Examination Survey, PIR = ratio of family income to poverty.

### 3.2. Association between female hormone intake and CircS

The results of the weighted logistic regression are shown in Table 3. In the unadjusted Model 1, the intake of female hormones did not exhibit a significant association with CircS (odds ratio [OR] = 1.08, 95% confidence interval [CI]: 0.93–1.26, *P* = .290). After adjusting for age, race, education level, marital status, and PIR in Model 2, the OR increased to 1.46 (95% CI: 1.22–1.74, *P* < .001). Further adjustments in Model 3, which included BMI, alcohol use, smoking, heart failure, coronary heart disease, and stroke, resulted in an OR of 1.47 (95% CI: 1.20–1.80, *P* < .001). This indicates that female hormone intake is independently associated with a 47% increase in the risk of CircS.

**Table 3:** Association between female hormone intake and circadian syndrome.

	N	OR (95% CI)	*P*
Model 1	7279	1.08 (0.93–1.26)	.29
Model 2	7279	1.46 (1.22–1.74)	<.001
Model 3	7279	1.47 (1.20–1.80)	<.001
Sensitivity analysis	6580	1.43 (1.16–1.77)	<.001

Model 1 adjusted no factor. Model 2 adjusted age, race, education level, marital status, and PIR. Model 3 adjusted age, race, education level, marital status, PIR, BMI, alcohol use, smoking, heart failure, coronary heart disease, and stroke. Sensitivity analysis adjusted age, race, education level, marital status, PIR, BMI, alcohol use, and smoking.

BMI = body mass index, CI = confidence interval, OR = odds ratio, PIR = ratio of family income to poverty.

### 3.3. RCS analysis

**Figure 2.**
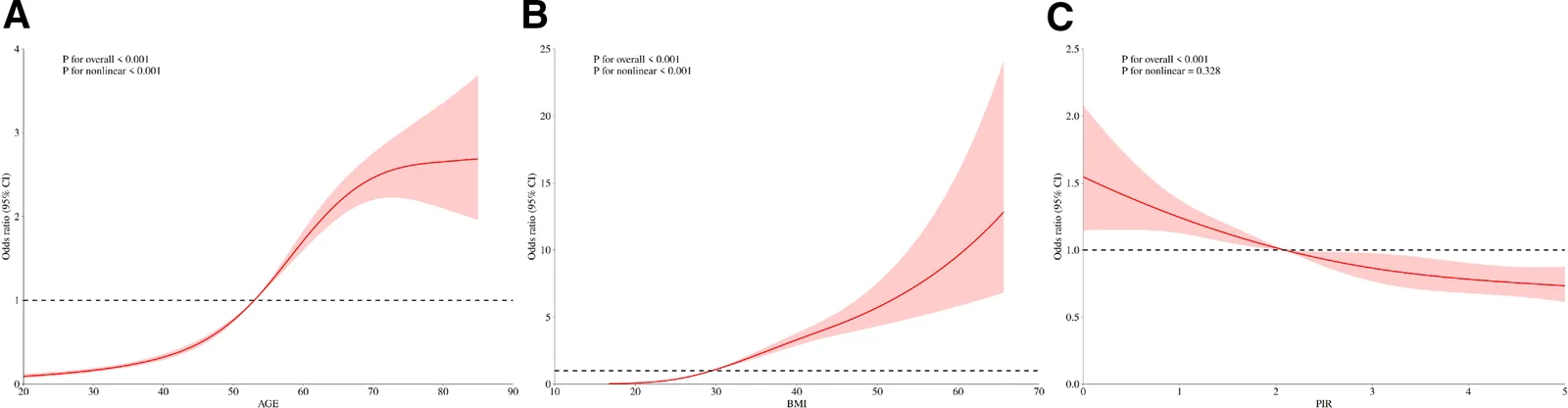
RCS curves for the associations of the age (A), BMI (B), PIR (C) with CircS. BMI = body mass index, CI = confidence interval, CircS = circadian syndrome, PIR = ratio of family income to poverty, RCS = restricted cubic spline.

RCS and threshold effect testing identified significant nonlinear relationships between both age and BMI with CircS (*P* < .001; Fig. 2A and B). The results of the threshold effect analysis are presented in Table 4. The age inflection point was determined to be 61.0 years. Below this threshold, each additional year of age corresponded to an 8% increase in the risk of CircS (OR = 1.08, 95% CI: 1.08–1.09, *P* < .001); beyond 61.0 years, the association attenuated considerably but remained statistically significant, with a 2% increase in risk per year (OR = 1.02, 95% CI: 1.01–1.04, *P* = .005). For BMI, the inflection point was 29.9 kg/m^2^. Below this value, each unit increase in BMI was linked to a 33% increase in risk (OR = 1.33, 95% CI: 1.29–1.37, *P* < .001); above 29.9 kg/m^2^, the risk increased by 8% (OR = 1.08, 95% CI: 1.07–1.10, *P* < .001). For PIR, RCS analysis indicates a notable linear trend (*P* for nonlinear = .328, Fig. 2C).

**Table 4:** Threshold effect analysis of age and body mass index with circadian syndrome.

Variable	OR (95% CI)	*P*
Age		
<61.0 yrs	1.08 (1.08–1.09)	<.001
≥61.0 yrs	1.02 (1.01–1.04)	.005
	*P* for likelihood test	<.001
BMI		
<29.9 kg/m^2^	1.33 (1.29–1.37)	<.001
≥29.9 kg/m^2^	1.08 (1.07–1.10)	<.001
	*P* for likelihood test	<.001

BMI = body mass index, CI = confidence interval, OR = odds ratio.

### 3.4. Sensitivity analysis

To further assess the reliability of the findings, a sensitivity analysis was performed by excluding participants with a history of heart failure, coronary heart disease, or stroke, followed by reevaluation using the fully adjusted model (Table 3). This analysis included 6580 women without cardiovascular comorbidities after excluding n = 251 with heart failure, n = 175 with coronary heart disease, and n = 273 with stroke. The association between female hormone intake and CircS remained significant (OR = 1.43, 95% CI: 1.16–1.77, *P* < .001), consistent with the main analysis results (OR = 1.47, 95% CI: 1.20–1.80, *P* < .001). This suggests that the primary findings were not substantially influenced by the presence of comorbidities.

### 3.5. Subgroup analysis

**Figure 3.**
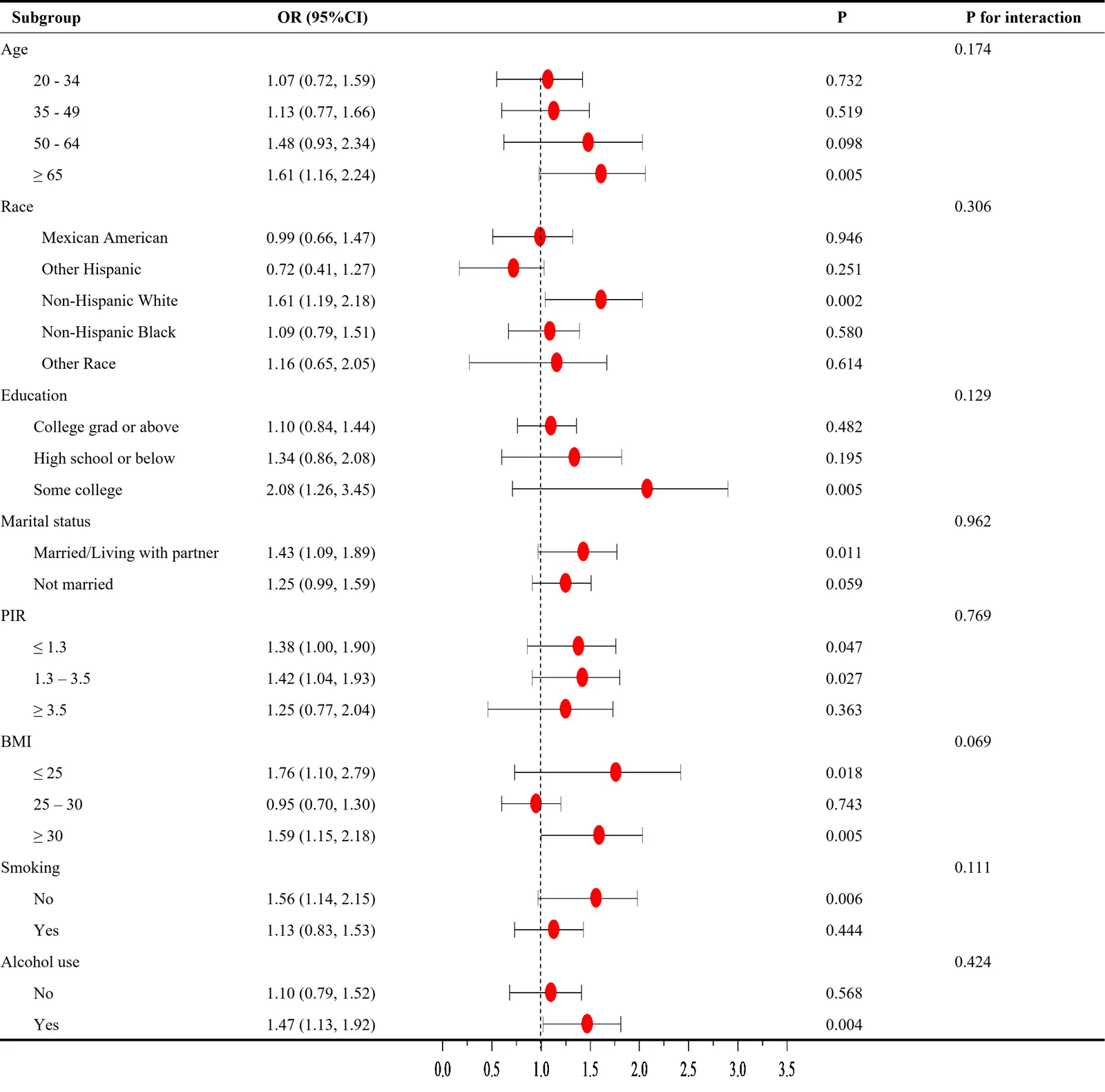
Subgroup analysis of the associations between female hormone intake and CircS. BMI = body mass index, CI = confidence interval, CircS = circadian syndrome, OR = odds ratio, PIR = ratio of family income to poverty.

The subgroup analysis results are shown in Figure 3. All *P* values for interaction were > .05, suggesting no significant variation in the association across different population characteristics. The association was statistically significant among those aged ≥ 65 years, non-Hispanic Whites, those with some college education, PIR < 3.5, people with obesity (BMI ≥ 30), nonsmokers, and alcohol users.

## 4. Discussion

This study, utilizing data from 7279 female participants in the NHANES 2005–2018, represents the first systematic evaluation of the relationship between female hormone intake and CircS. The principal findings are as follows: female hormone intake is independently correlated with an elevated risk of CircS (OR = 1.47, *P* < .001) and age and BMI demonstrate significant nonlinear associations with CircS, with inflection points identified at 61.0 years and 29.9 kg/m^2^, respectively.

Baseline characteristics indicated that the CircS group, in comparison to the non-CircS group, exhibited a higher mean age (56.69 vs 43.05 years), reduced educational level, a smaller proportion of married or cohabiting individuals, a lower PIR, an elevated BMI, and increased rates of smoking and cardiovascular comorbidities (all *P* < .05). These disparities suggest a generally poorer socioeconomic and metabolic profile among CircS patients, necessitating the adjustment for these confounders in subsequent analyses.

Notably, female hormone intake was not significantly associated with CircS in the unadjusted model (OR = 1.08, *P* = .290). However, after full adjustment, the association became significant and stronger (OR = 1.47, *P* < .001). This change indicates that confounders, including age and socioeconomic status, obscured the true effect of hormones in the unadjusted model. This observation aligns with the baseline differences previously described. Estrogen potentially affects the development of CircS through various biological mechanisms. Initially, estrogen regulates the expression of clock genes, including CLOCK, BMAL1, PER1/PER2, and CRY1/CRY2, within the suprachiasmatic nucleus via nuclear receptors (ERα and ERβ) and the membrane receptor (GPER1), thereby directly disrupting circadian rhythms.^[16,17]^ Research involving animals has shown that both a deficiency in estrogen and exposure to exogenous estrogen can modify the rhythmic expression of core clock genes, resulting in a disruption of metabolic rhythms.^[18]^ Second, exogenous hormones, especially when administered orally, influence lipid metabolism via the hepatic first-pass effect.^[19]^ Oral estrogen can markedly increase triglyceride levels and alter the expression of inflammatory factors. Third, the regulation of sleep and mood by estrogen may indirectly elevate the risk of CircS by affecting the components of short sleep duration and depression.^[20]^

The RCS analysis identified a significant age inflection point at 61.0 years, delineating 2 distinct phases of risk accumulation. Prior to age 61, the odds of CircS increased sharply by 8% annually, likely due to the progressive decline in circadian and metabolic regulation during the premenopausal and early postmenopausal periods.^[21,22]^ Beyond age 61, the risk continued to rise, albeit at a reduced rate of 2% per year. This deceleration may be attributed to several factors: the stabilization of the postmenopausal hormonal environment, the establishment of new metabolic equilibria, and a selective survival effect, wherein women most susceptible to CircS-related morbidity may have been removed from the population at younger ages. The continued statistically significant positive association beyond 61 years indicates that aging maintains a modest influence on circadian–metabolic risk even in later life, highlighting the necessity for ongoing surveillance beyond midlife. The BMI inflection point of 29.9 kg/m^2^ closely aligns with the WHO obesity threshold of ≥ 30 kg/m^2^. Below this threshold, each unit increase in BMI was associated with a 33% increase in risk, while above it, the risk increase was limited to 8%.

The sensitivity analysis, excluding participants with a history of heart failure, coronary heart disease, or stroke, confirmed that the association remained robust. This finding indicates that the primary results were not significantly affected by the presence of cardiovascular comorbidities.

This study elucidates significant clinical and public health implications. The period before age 61, particularly during perimenopause and early postmenopause, is a critical phase for the prevention of CircS. During this time, clinicians should prioritize annual screenings of waist circumference, blood glucose levels, blood pressure, and lipid profiles, in addition to proactive assessments of sleep quality and depressive symptoms. The BMI inflection point of 29.9 kg/m^2^ closely aligns with the clinical obesity threshold. The steeper gradient observed below this threshold suggests a rapid escalation in metabolic risk as BMI nears 30 kg/m^2^.^[23]^ These findings indicate that individuals with a BMI approaching 28 to 29 kg/m^2^ should be prioritized for intensive metabolic monitoring and lifestyle intervention, rather than delaying until the formal onset of obesity.

This study has certain limitations. First, the cross-sectional design limits the ability to infer causality. Second, the assessment of female hormone intake relied on self-reported data, which may introduce recall and reporting biases. Third, the RCS analysis of PIR revealed a significant overall linear association with CircS (*P* for overall < .001), while a nonlinear relationship was not supported (*P* for nonlinear = .328). The limited sample size in the extreme low-income categories may have diminished the statistical power to detect potential nonlinearity or threshold effects. Sparse data bias at the lower end of PIR cannot be excluded,^[24]^ necessitating cautious interpretation of effect estimates in this range. Consequently, the precise dose–response shape of the PIR–CircS association requires further investigation in cohorts with sufficient representation of low-income populations. Additionally, subgroup analysis did not indicate significant effect modification by PIR on the relationship between female hormone intake and CircS.

## 5. Conclusion

This study established that the intake of female hormones is independently correlated with an elevated risk of CircS, as indicated by an odds ratio of 1.47 (95% CI: 1.20–1.80, *P* < .001). Age and BMI demonstrated nonlinear relationships with CircS, with critical points identified at 61.0 years and 29.9 kg/m^2^, respectively. These results contribute novel insights into the relationship between female reproductive hormone exposure and circadian metabolic health, providing a scientific foundation for the personalized prevention of CircS, particularly in middle-aged women and individuals with higher BMI.

## Acknowledgments

We extend our sincere gratitude to all participants, staff, and investigators of the NHANES for their generous allocation of time, which facilitated the completion of this study and many others.

## Author contributions

**Investigation:** Wei Wang, Zhiqin Ding.

**Methodology:** Hao Wu.

**Software:** Hongqin Ding, Zhiqin Ding.

**Supervision:** Yucheng Liu.

**Visualization:** Dan Zheng, Zhiqin Ding.

**Writing – original draft:** Zhiqin Ding.

**Writing – review & editing:** Weifu Xiong.
